# Bioconversion of C1-gases by mixotrophic co-cultures fermentation with *C. carboxidivorans* and *C. beijerinkii*

**DOI:** 10.1186/s40643-025-00881-w

**Published:** 2025-05-26

**Authors:** Marina Fernández-Delgado, Pedro Enrique Plaza, M. Teresa García-Cubero, Susana Lucas, Mónica Coca, Juan Carlos López-Linares

**Affiliations:** 1 Department of Chemical Engineering and Environmental Technology, University of Valladolid, Dr. Mergelina S/N, 47011 Valladolid, Spain; 2 Department of Chemical Engineering and Environmental Technology, Institute of Sustainable Processes, University of Valladolid, Dr. Mergelina S/N, 47011 Valladolid, Spain

**Keywords:** C1-gases conversion, Mixotrophic fermentation, Fe^0^, Co-culture, *Clostridia *spp.

## Abstract

The development of newfangled bioprocess strategies for the capture of C1-gases (CO and CO_2_) and their bioconversion into valuable products is currently one of the main focuses of research in order to achieve a more resilient world. This work analyses the viability of the co-culture *C. carboxidivorans* and *C. beijerinkii* to produce bioproducts (bioalcohols and organic acids) in mixotrophic conditions. In this way, the bioconversion of C1 gases (CO and CO_2_), in the presence of Fe^0^, using mixotrophic co-culture fermentation by *C. carboxidivorans* and *C. beijerinkii*, was evaluated, analyzing the influence of the ratio between both microorganisms, the pH, and the presence of Fe^0^. As a result, up to 7 g/L of butanol were achieved at pH 7, 12.5 g/L Fe^0^, and using a 1:1 ratio of *C. carboxidivorans*: *C. beijerinkii*, also improving the production of ethanol, acetic acid, and butyric acid as compared to individual culture fermentations. Finally, the operation in a bioreactor, comparing discontinuous and continuous gas feeding operation modes, was also studied, with better C1-gases utilization and overall fermentation efficiency (7 vs 4.6 g/L butanol) in continuous gas operation mode.

**Supplementary Information:**

The online version contains supplementary material available at 10.1186/s40643-025-00881-w.

## Highlights

- Mixotrophic co-cultures by *C. carboxidivorans* and *C. beijerinkii* in presence of Fe^0^
- Influence of microorganisms ratio, pH, and Fe^0^ presence in mixotrophic co-cultures
- 7 g/L butanol (pH 7, 12.5 g/L Fe^0^) by *C. carboxidivorans*:*C. beijerinkii* (1:1)
- Acceptable C1-gases use in biorreactor co-culture with continuous gas feeding

**Graphical Abstract:**

**Supplementary Information:**

The online version contains supplementary material available at 10.1186/s40643-025-00881-w.

## Graphical abstract

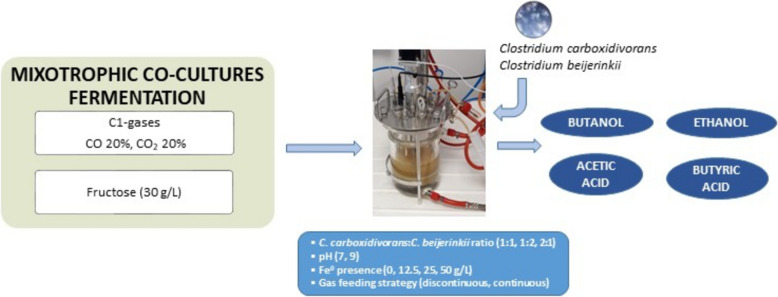

## Introduction

Due to the continuous use of fossil fuels, deforestation and other human activities, a great amount of carbon dioxide (CO_2_), known as one-carbon (C1) gases, are generated (Sivalingam et al. 2022). In this way, more than 36 billion tons of CO_2_ were emitted into the atmosphere in 2023, which resulted in 420 ppm of atmospheric CO_2_ concentration (NOAA 2023). Moreover, emissions are not expected to be reduced in the short term (Salehizadeh et al. 2020). C1 gases are considered one of the main contributors to environmental damage and climate change (Wu et al. 2023). On the other hand, a typical composition of a waste gas originating from the industrial combustion process could be CO (20–35%), CO_2_ (20–30%), and N_2_ (50–60%) (Molitor et al. 2016). Therefore, technologies which are able to use C1 gases sustainably are necessary in order to fight against climate change (Schulz et al. 2023). In this context, biological processes that are considered economic and sustainable are being explored (Gupta et al. 2022).

Biological processes could be able to produce a wide variety of main products and by-products from C1 gases, such as biofuels (ethanol and butanol), medium chain acids (acetic, lactic, formic and butyric acids, among others), 2,3-butanediol, or acetoin, among others (Zhang et al. 2016a). *Clostridium* spp. strains are acetogenic microorganisms able to anaerobically become C1-gases in organic acids and alcohols by the Wood-Ljungdahl pathway (WLP) (Arslan et al. 2021). Of these, *Clostridium carboxidivorans* is worth noting, as it has been widely used in the literature to metabolize C1 gases (Table 1) (Fernández-Naveira et al. 2017b, 2019; Sun et al. 2018; Benevenuti et al. 2020, 2021; Cheng et al. 2022; Bayar et al. 2022; Antonicelli et al. 2023; Thunuguntla et al. 2024a, b; Lanzillo et al. 2024). *C. carboxidivorans* uses energy and carbon to grow from C1 gases, initially producing a mixture of organic acids (acetic and butyric acids, among others) for energy conservation and cell material synthesis; later accumulating the corresponding C2 and C4 alcohols (ethanol and butanol) (Liu et al. 2014; Fernández-Naveira et al. 2017a, c). In order to metabolize CO_2_, *C. carboxidivorans* needs an external supply of energy (usually an H_2_ gas) capable of reducing the CO_2_ to formate in the first step of the methyl branch of the WLP; or capable of reducing CO_2_ to CO in the carbonyl branch of the WLP (Schuchmann and Müller 2014). However, the external addition of H_2_ is not advisable, as it has a low sustainability and high bioprocessing efficiency cost. In order to overcome these drawbacks, the use of Zero Valent Iron (ZVI (Fe^0^)), which is a non-toxic, abundant and cheap compound able to generate H_2_ under anaerobic conditions, could be a greener and more interesting option as a potential electron donor for CO_2_ bioconversion (Hwang et al. 2019; Bayar et al. 2022). It is worth noting that the use of C1-gases as substrates in fermentation processes often falls short of achieving the theoretical product yields predicted by the WLP (Liew et al. 2016). In recent years, novel fermentation strategies, such as the use of mixotrophic substrates, comprising a blend of heterotrophic and autotrophic substrates, have emerged as promising avenues to enhance the production of alcohols and organic acids from C1-gases (Vees et al. 2022; Yang et al. 2022). This mixotrophic approach has the potential to address the limitations of both sugar fermentations (carbon loss due to CO_2_ production) and gas fermentations (low productivity and feedstock solubility in liquids) (Dash et al. 2016; Jiang et al. 2021). In this way, the sugar presence enhances the production of such crucial intermediates as pyruvate and acetyl-CoA, improving the overall efficiency of the fermentation process (Vees et al. 2022; Yang et al. 2022). Consequently, mixotrophic fermentation could lead to the development of adaptable and highly efficient production platforms.

**Table 1:** Comparative studies about metabolizing C1 gases by *Clostridium carboxidivorans*

Microorganism	Tª (ºC)	Agitation (rpm)	Gas composition	By-products conc. (g/L)				References
				Acetic acid	Butyric acid	Ethanol	Butanol	
*C. carboxidivorans* P7	37	125	H_2_:CO_2_:N_2_ (60:20:20)	8.0	0.5	4.0	0.6	Thunuguntla et al. (2024a)
*C. carboxidivorans* P7 (DSM 15243)	33	250	CO:CO_2_:H_2_:N_2_ (30:10:20:40)	2.5	0.5	5.9	2.1	Fernández-Naveira et al. (2019)
*C. carboxidivorans* P7 (ATCC BAA-624)	37	0	CO:H_2_:CO_2_ (40:30:30)	1–3	0.1–0.4	2.5–3.5	0.6–1.1	Sun et al. (2018)
*C. carboxidivorans* DSM 15243	37	150	CO:H_2_:CO_2_:N_2_:methane (25: 43.9: 10.02: 10.05: 11.01)	3.6	–	2.3	0.7	Benevenuti et al (2020)
*C. carboxidivorans* P7 (DSM 15243)	37	0	CaCO_3_, glucose, exogenous electron, biochar	3.0	2.7	0.5	0.3	Cheng et al. (2022)
*C. carboxidivorans* DSM 15243	30	150	CO_2_ (100%)	2.2	–	–	–	Bayar et al. (2022)
*C. carboxidivorans* P7 (DSM 15243)	37	0	CO:H_2_:CO_2_:N_2_ (56:20:9:15)	1.0	0.5	0.7	0.3	Antonicelli et al. (2023)
			H_2_:CO_2_ (4:1)	0.9	0.3	0.4	0.5	
*C. carboxidivorans* DSM 15243	35	250	CO (100%)	0.6	0.2	0.9	0.2	Lanzillo et al. 2024
			CO:CO_2_:H_2_ (65:10:25)	0.7	0.3	1.0	0.4	
*C. carboxidivorans* DSM 15243	37	150	CO:CO_2_:N_2_ (20:20:60)	0.2	0.8–1.9	1.1–1.4	–	This study

On the other hand, *C. beijerinckii* is known as being mainly responsible for acetone-butanol-ethanol (ABE) fermentation (Wang et al. 2019; Qi et al. 2019). Unlike *C. carboxidivorans*, *C. beijerinckii* is not able to metabolize C1 gases, but rather uses carbohydrates and organic acids (like acetic and butyric acids) as the carbon source. However, as with *C. carboxidivorans*, *C. beijerinckii* generate alcohols in a two-step process: firstly, with exponential bacterial growth and the production of different organic acids; and then with a second step, where the acids are turned into ABE (Cai et al. 2016; Xue and Cheng 2019; López-Linares et al. 2021). Therefore, the use of co-culture with both *C. carboxidivorans* and *C. beijerinckii* can be considered a great option, where *C. carboxidivorans* can utilize C1-gases (CO and CO_2_) to generate such organic acids as acetic and butyric acids; while *C. beijerinckii* can convert these acids into butanol without consuming gases. This synergy allows for a continuous production cycle, optimizing substrate utilization and enhancing productivity.

The main objective of this work is to evaluate the viability of C1 gases (CO:CO_2_:N_2_, 20:20:60) bioconversion to valuable biofuels (alcohols) and organic acids, using mixotrophic co-culture fermentation by the acetogenic bacteria *C. carboxidivorans* and *C. beijerinckii*. Moreover, the influence of the ratio of the microorganisms, the initial fermentation pH, and the presence of Fe^0^ was also analyzed in terms of fermentation efficiency. Scaling up to a stirred tank bioreactor, comparing discontinuous and continuous gas feeding operation modes, was also studied. To the best of our knowledge, this is the first work where the bioconversion of C1 gases (CO:CO_2_:N_2_, 20:20:60) by the co-cultures *C. carboxidivorans* and *C. beijerinckii*, and in the presence of Fe^0^ as potential electron donor for CO_2_ bioconversion, has been studied.

## Materials and methods

### Microorganism and culture media

The microorganisms used in this study were *C. carboxidivorans* DSM 15243 and *C. beijerinckii* DSM 6422, from the German collection of microorganisms (DSMZ, Leibniz, Germany). Firstly, lyophilized cells were inoculated into a DSMZ liquid medium, grown at 35 °C, 150 rpm and 48 h in an orbital shaker (Optic Ivymen Systems, Comecta, Spain), and finally stored as glycerol stock (40% v/v) at − 80 °C until further use.

Both strains were grown in 100 mL septum bottles, with a rubber septum, 50 mL being the working volume used. A mixture of C1 gases (CO:CO_2_:N_2_, 20:20:60) was used as headspace for *C. carboxidivorans*, which is considered as the typical composition of a waste gas from an industrial combustion process (Molitor et al. 2016); while pure N_2_ was employed for *C. beijerinckii*. The cells were grown in a rotary shaker at 35 °C and 150 rpm for 48 h (*C. carboxidivorans*), or 24 h (*C. beijerinckii*).

The composition of the liquid culture medium used for *C. carboxidivorans* was DSMZ medium (per liter of distilled water): 10 g yeast extract, 5 g of Trypticase peptone (BD BBL), 5 g of meat peptone (pepsin-digested), 0.5 mL resazurin (from a stock solution of 0.5 g/L), 40 mL of salt solution, and 50 mL of secondary solution. The salt solution contained the following (per liter of distilled water): 0.25 g of CaCl_2_·2H_2_O, 0.5 g of MgSO_4_·7 H_2_O, 1 g of K_2_HPO_4_, 1g of KH_2_PO_4_, 2g of NaCl, and 10 g of NaHCO_3_. The secondary solution consisted of the following (per liter of distilled water): 150 g of glucose, 20 g of Na_2_CO_3_, and 10 g of cysteine HCl·H_2_O. The medium used for *C. beijerinckii* was RCM (Reinforced Clostridial Medium) (Sigma-Aldrich, Spain).

Both the *C. carboxidivorans* and *C. beijerinckii* media (except for salt and secondary solutions for *C. carboxidivorans*) were sterilized at 121 °C for 15 min in septum bottles (previously flushed with nitrogen into the liquid). In contrast, the salt and secondary solutions for *C. carboxidivorans* were sterilized by filtration, using 0.2 μm cellulose nitrate filters (Sartorius 254 stedim Biotech, Göttingen, Germany).

### *C. carboxidivorans* and *C. beijerinckii* co-cultures in sealed bottles

Co-culture fermentation tests by *C. carboxidivorans* and *C. beijerinckii* were carried out, studying different factors: the ratio between both microorganisms (1:1, 1:2, 2:1 (v/v)), being these levels selected according to Du et al. (2020); pH (7 and 9), based in the results obtained in previous control experiments by *C. carboxidivorans*; and use or not of Fe^0^ (12.5, 25 and 50 g/L), being these concentrations chosen according to Bayar et al. (2022).

The culture medium used was that described for *C. carboxidivorans* in "Microorganism and culture media" setion, with the initial pH value (7 or 9) adjusted in each case. Fructose (30 g/L), from a stock solution of 500 g/L (previously sterilized by filtration), was added initially (t = 0) or at t = 24 h of fermentation. Fe^0^ (12.5, 25 and 50 g/L) was added in some co-culture tests (ratio 1:1 and pH 7) at the start of fermentation. The co-culture fermentation tests were carried out in 100 mL sealed bottles (previously sterilized at 121 °C for 15 min) equipped with a rubber septum, using 50 mL as working volume. A mixture of CO:CO_2_:N_2_ (20:20:60) was introduced in the headspace at the start of fermentation, which was replaced on the fourth day to liberate overpressure in the bottles. The inoculum loading used for both microorganisms was 10% (v/v), with *C. carboxidivorans* being inoculated at the beginning of fermentation (t = 0) and *C. beijerinckii* at t = 24 h of the process. The co-culture fermentation was carried out in an orbital shaker at 35 °C and 150 rpm.

Control of the simple culture by *C. carboxidivorans* in autotrophic and heterotrophic conditions were also conducted, at different initial pHs (5–9). pH values were selected according to other works from the literature (He et al. 2022; Fernández-Blanco et al. 2023). In autotrophic fermentation, a mixture of CO:CO_2_:N_2_ (20:20:60) was used as a substrate; while in heterotrophic fermentation, fructose (30 g/L) was employed. The same medium employed in the co-culture essays (*C. carboxidivorans* medium) was used under anaerobic conditions (using the mixture CO:CO_2_:N_2_ (20:20:60) in autotrophic fermentations and pure N_2_ in heterotrophic fermentations). Control of the simple culture by *C. beijerinckii* was also carried out, using the same medium employed in the co-culture essays (*C. carboxidivorans* medium), with pH 7, 30 g/L of fructose (at t = 0), 50 g/L of Fe^0^, and CO:CO_2_:N_2_ (20:20:60) mixture. Both simple cultures were performed under the same conditions of temperature and agitation as described for the co-culture essays, using an inoculum loading of 10% v/v, and no pH control during the fermentation.

Liquid samples were taken every 24 h, centrifuged (13,500 rpm, 10 min), and analyzed for their content in fructose and fermentation products (ethanol, butanol, and acetic and butyric acids). A 1 mL gaseous sample was also taken from headspace every 24 h, and their composition, in terms of CO, CO_2_, and N_2_ concentration, was analyzed.

All fermentation tests were performed in duplicate.

### *C. carboxidivorans* and *C. beijerinckii* co-cultures in bioreactor

Co-culture fermentation tests by *C. carboxidivorans* and *C. beijerinckii* were also carried out in a 2L Biostat B Plus bioreactor (Sartorius®) at 35 ºC and 50 rpm, using the medium described previously for the serum bottles (at pH 7) and 1 L as working volume. An inoculum loading of 10% (v/v), with a ratio of 1:1, was used for both microorganisms, *C. carboxidivorans* and *C. beijerinckii* being inoculated at t = 0 and t = 24 h of the process, respectively. Fructose (30 g/L) and Fe^0^ (12.5 g/L) were added at the start of the fermentation. A mixture of CO:CO_2_:N_2_ (20:20:60) was introduced in the bioreactor headspace, with two gas feeding strategies being studied: discontinuous gas feeding operation mode, with all the gas being displaced from the headspace at t = 0 using the CO:CO_2_:N_2_ (20:20:60) mixture; and continuous gas feeding operation mode, using 50 mL/min of gas flow of the CO:CO_2_:N_2_ (20:20:60) mixture.

Liquid samples were taken during fermentation, centrifuged (at 13,500 rpm for 10 min) and analyzed for their content in fructose and fermentation products (ethanol, butanol, and acetic and butyric acids). A 1 mL gaseous sample was also taken from headspace, and the composition was analyzed in terms of CO, CO_2_, and N_2_ concentration.

### Analytical methods

The content of fructose and fermentation products (such as ethanol, butanol, and acetic and butyric acids, among others) in the liquid phase was analyzed by High-Performance Liquid Chromatography (HPLC), using a refractive index detector (Waters 2414), an Aminex HPX-87H column (at 30 °C (solvents) or 60 °C (fructose, organic acids), and 0.01 N H_2_SO_4_ (0.6 mL/min) as the mobile phase.

An 8860 GC gas chromatograph (GC, Agilent Technologies, Spain), equipped with a thermal conductivity detector (TCD), was used to determine the gas composition in the gaseous samples, employing helium as the carrier gas. The GC was fitted with a 15-m HP-PLOT Molecular Sieve 5A column (ID, 0.53 mm; film thickness, 50 μm) at 45 °C, with 250 °C as the detector temperature.

The optical density (OD) at 600 nm was measured using a spectrophotometer (Uvmini-1240, Shimazu Suzhou Wfg., Kyoto, Japan) to analyze the cell concentration in the liquid samples.

All analytical determinations were carried out in triplicate, and the average results are shown.

### Data analysis

The statistical software R (version 4.2.2.—Innocent and Trusting—2022) was employed to investigate the influence of process variables on the fermentation process and explore significant differences between them. This analysis included carbon balances and temporal visualizations to assess how these substrates and products changed throughout the experiments. Carbon balances were determined calculating the product of moles of each compound involved in fermentation by its carbon atoms number, at the beginning and at the end of fermentation. CO and CO_2_ moles were calculated considering the ideal gas law.

## Results and discussion

Heterotrophic (using fructose as a substrate) and autotrophic fermentations (using a C1-gases mixture as substrate) by *C. carboxidivorans* were planned to understand its behavior at different initial pH levels (5, 6, 7, 8, and 9), which were not controlled during the experimental run. In this way, different pH values were tested, since this work aimed to produce organics acids by *C. carboxidivorans* in a first stage using co-cultures, which can be later metabolized by *C. beijerinckii* in a second stage in co-cultures.

The initial pH of the fermentation medium significantly influences product formation; for instance, a highly basic initial pH inhibits heterotrophic fermentation (Figure S1). However, for pH 5–8, *C. carboxidivorans* is able to metabolize about 4–5 g/L fructose in fermentations under heterotrophic conditions, which could be enough for the growth of the microorganism (3.5–6 OD of biomass), as well as for the production of metabolites (ethanol, 2000–3000 mg/L; acetic acid, 1000–1500 mg/L; and butyric acid, 1600–2200 mg/L). Conversely, in autotrophic fermentation, the microorganism efficiently uses C1 gases as its sole carbon source, mainly producing more butyric acid (1.8–2 g/L) at more basic initial pH levels and operating in the acidogenic phase (Figure S2) (Fernández-Blanco et al. 2023). Liu et al. (2014) also reported high metabolites production (6 g/L ethanol and 1.1 g/L butanol) in the autotrophic fermentation at high basic pH (pH 8) by *Alkalibaculum bacchi* CP15, using as syngas the mixture CO/CO_2_/H_2_/N_2_ (20/15/5/60). These good results achieved at high initial basic pH could be due to the decrease in pH that takes place initially (t = 0) when C1-gas or syngas is purged in fermentation medium due to the presence of CO_2_ from the C1-gas or syngas, and then, due to the acids production (acetic and butyric acids) during the fermentation process (Liu et al. 2014). In the review of Fernández-Blanco et al. (2023), other authors have also been reported to carry out successfully autotrophic fermentations at high pHs from C1-gases. So for example, from pure CO_2_ by *C. aceticum* (DSM 1496) and *C. carboxidivorans* (DSM 15243) at pH 8.5 (Bayar et al. 2022); from syngas by *C. aceticum* at pH of about 7 (Arslan et al. 2021; Fernández-Blanco et al. 2022); from a CO-H_2_ mixture at pH 6.9 by *Moorella thermoacetica* (Takemura et al. 2021); from CO/H_2_-CO mixture at pH 7.7 by *Eubacterium limosum* (Litty and Müller 2021); and from syngas at pH 7.5 by *C. aceticum* + *C. kluyveri* (Fernández-Blanco et al. 2022), among others.

### *C. carboxidivorans* and *C. beijerinckii* co-cultures in sealed bottles

#### Study of co-culture ratio

Three different ratios of *C. carboxidivorans* to *C. beijerinckii* were proposed for the study: 1:1, 1:2, and 2:1. Based on previous results from autotrophic fermentations with *C. carboxidivorans*, the process was conducted at initial pH 9 as this pH level has been shown to maximize the concentration of acids.

**Fig. 1:**
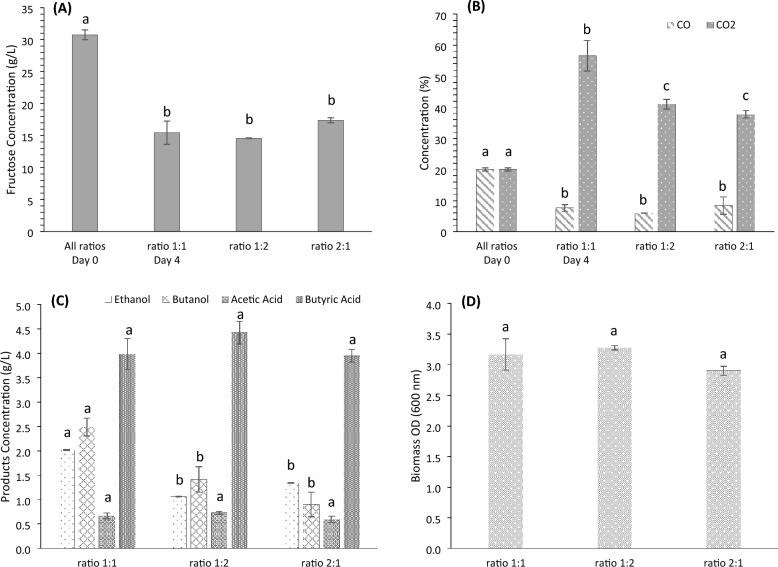
Fermentation co-cultures results by *C. carboxidivorans* (t = 0) and *C. beijerinckii* (t = 24h) for different microorganism ratios (1:1, 1:2, 2:1). **A** Fructose Concentration (g/L), **B** Gas Concentrations (CO and CO_2_) (%), **C** Product Concentrations (Ethanol, Butanol, Acetic Acid, and Butyric Acid) (g/L), and **D** Biomass OD (600 nm). The results are at the 4th day related to the maximum product concentration obtained. Each letter denotes statistically significant differences for the corresponding compound

Figure 1 summarizes the results obtained in this case. Regarding the fructose uptake (Fig. 1A), the consumption ranged from 46 to 58%, stabilizing after 4 days of fermentation. However, these results did not show significant differences (*p* > 0.05) among the tested ratios. It is most likely that this consumption is attributed to *C. beijerinckii*, since *C. carboxidivorans* consumes almost no fructose and focuses more on CO consumption. CO consumption and CO_2_ production are shown in Fig. 1B. In this case, we can see that, for the ratios studied, CO consumption is similar (around 50% of the initial CO) with no significant differences between them (*p* > 0.05). As for CO_2_, it has been generated instead of consumed, reaching up to 60% in the 1:1 ratio. This may be because *C. beijerinckii* produces CO_2_ within its metabolic pathway (Yang et al. 2018) and, as no H_2_ is used which could make *C. carboxidivorans* consume it (Fernández-Blanco et al. 2023), the percentage of CO_2_ in the headspace increases.

Regarding the products obtained, Fig. 1C demonstrates that the co-cultures produced ethanol, butanol, acetic acid, and butyric acid. The maximum ethanol concentration was observed at a ratio of 1:1 (~ 2 g/L), which was significantly different (*p* < 0.05) from the other ratios. Similarly, butanol production reached its peak concentration (2.5 g/L) at a 1:1 ratio. In contrast, acetic acid production was consistent across all three ratios (~ 0.7 g/L). For butyric acid, although the highest concentration (4.4 g/L) was achieved at a 1:2 ratio, there were no significant differences (*p* > 0.05) compared to the other ratios studied (~ 4 g/L). When comparing these results with those obtained using *C. carboxidivorans* in autotrophic fermentation, it is evident that the co-culture with a mixotrophic substrate (fructose and gases) yields double the amount of ethanol compared to autotrophic fermentation at the same initial pH (2 g/L vs. 1 g/L). Additionally, the acetic acid concentration was improved, as *C. carboxidivorans* with a gaseous substrate produced only 0.1 g/L of acetic acid, whereas the co-culture achieved 0.7 g/L. Notably, the butyric acid concentration is almost 2.5 times higher in the mixotrophic co-culture fermentation compared to the autotrophic fermentation (4.4 g/L vs. 1.8 g/L). Finally, butanol (2.5 g/L) in the co-culture fermentation is significant, since this metabolite was not produced with *C. carboxidivorans* alone. On the other hand, compared to co-culture fermentation, cultures by only *C. beijerinckii* also yielded much lower concentrations of butyric acid (0.2 vs 4.4 g/L), ethanol (0.4 vs 2 g/L), acetic acid (0.4 vs 0.7 g/L), and similar butanol levels (about 2.5–3 g/L). These improvements can be attributed to two factors. First, two carbon sources benefit each microorganism individually (C1-gases for *C. carboxidivorans* and fructose for *C. beijerinckii*). Second, the co-culture appears to exhibit a good synergy between these two microorganisms, as they complement each other in resource utilization, leading to an increased production of the fermentation products of interest (Du et al. 2020).

Finally, the biomass OD (Fig. 1D) was similar across all ratios (ranging from 2.9 to 3.3) and did not show significant differences (*p* > 0.05). This consistency in biomass might be due to the efficient division of labor between the two microorganisms, allowing both to thrive, while not outcompeting each other for resources. The balanced growth could ensure that both microorganisms can maintain their metabolic activities effectively, resulting in a stable and productive fermentation process.

When comparing the obtained results with those reported in the literature, it is evident that the current study yields superior outcomes. For instance, Lanzillo et al. (2020) reported the production of 0.72 g/L of acetic acid, 0.09 g/L of butyric acid, 0.12 g/L of ethanol, and 0.03 g/L of butanol when working in small bottles at pH 5.75 with *C. carboxidivorans* and a mixture of CO and N_2_ as substrates. In another study, Fernández-Naviera et al. (2016) achieved concentrations of 0.4 g/L of ethanol, 0.9 g/L of acetic acid, and 0.2 g/L of butyric acid using only CO as the substrate at pH 5.75. Finally, Vees et al. (2022) used a mixotrophic substrate (20% CO, 10 g/L glucose) with continuous gas-feed and reached 5.7 g/L of ethanol, 2.6 g/L of butanol, 3.1 g/L of acetic acid, and 0.7 g/L of butyric acid. These comparisons suggest that using a co-culture (*C. carboxidivorans* and *C. beijerinckii*) and implementing a mixotrophic substrate (C1-gas and fructose) enhances the production of fermentation products. This improvement can be attributed to several factors. Firstly, as said before, the co-culture leverages the complementary metabolic capabilities of the two microorganisms. Secondly, the mixotrophic approach provides dual carbon sources, stimulating different metabolic pathways and enhancing overall metabolic activity. This dual substrate system likely prevents substrate limitation and maintains a steady supply of carbon for both microorganisms, leading to higher product concentrations (Jones et al. 2016; Vees et al. 2022). However, the different microorganism ratios did not significantly affect the products obtained or the consumption of the various substrates. Therefore, future work will utilize a 1:1 ratio between *C. carboxidivorans* and *C. beijerinckii*.

#### Influence of pH

While the previous section demonstrated that an initial pH 9 yielded favorable results, particularly for butanol and butyric acid production, the question remains whether adjusting the fermentation medium to a more basic initial pH is worthwhile. Therefore, this section analyzes the impact of the natural fermentation medium pH (pH 7) as compared to the adjusted medium pH (pH 9).

**Fig. 2:**
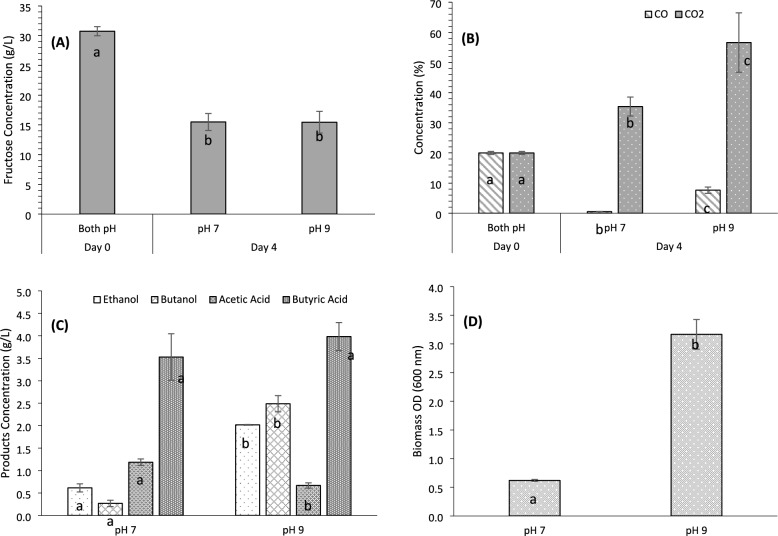
Fermentation co-cultures results for different pH (7, 9), by *C. carboxidivorans* (t = 0) and *C. beijerinckii* (t = 24h) at ratio 1:1. **A** Fructose Concentration (g/L), **B** Gas Concentrations (CO and CO_2_) (%), **C** Product Concentrations (Ethanol, Butanol, Acetic Acid, and Butyric Acid) (g/L), and **D** Biomass OD (600 nm). The results are at the 4th day related to the maximum product concentration obtained. Each letter denotes statistically significant differences for the corresponding compound

Figure 2 summarizes the results obtained. Regarding the mixotrophic substrate, Fig. 2A illustrates that fructose consumption was statistically similar (*p* > 0.05) under both conditions, decreasing from 30 g/L to approximately 15 g/L. However, regarding the autotrophic aspect, Fig. 2B indicates that at pH 7, the CO was almost entirely consumed, whereas at pH 9, only around half of the available CO was utilized, making these results statistically significant (*p* < 0.05). Moreover, as discussed in the previous section, CO_2_ is not consumed like CO due to overproduction by *C. beijerinckii*. However, it is noteworthy that, as shown in Fig. 2B, CO_2_ production at pH 7 is almost 40% lower than at pH 9, with significant differences between them (*p* < 0.05). Therefore, concerning substrate utilization, working with the natural pH (7) of the fermentation medium appears more advantageous.

Considering the products formed (Fig. 2C), there is a clear difference in the production of alcohols between the two pH levels studied. In this way, at pH 9, 2.2 g/L of ethanol and 2.5 g/L of butanol are obtained, compared to 0.6 g/L of ethanol and 0.3 g/L of butanol at pH 7. This means that at pH 9, between 70 and 90% more alcohols are produced than at pH 7, which is highly significant (*p* < 0.001). According to Fernández-Blanco et al. (2023), firstly a high pH period is needed to favour acetogenesis and bacterial growth; then, after the exponential growth phase and once acetic and butyric acids have been generated, pH decreases and solventogenesis is favoured, generating alcohols such etanol and butanol. However, this difference is not too big in the production of acids (Fig. 2C), because, at both pH 7 and pH 9, both *C. carboxidivorans* and *C. beijerinckii* are in the acidogenic phase (Branska et al. 2018). In the case of acetic acid, there are significant differences (*p* < 0.001) between the two pH levels, as pH 7 yields almost double the acetic acid (1.2 g/L) compared to pH 9 (0.7 g/L). On the other hand, this does not apply to butyric acid. The concentrations obtained range between 3.5 and 4 g/L. Still, as shown in Fig. 2C, the butyric acid concentrations have a high coefficient of variation (8%—15%), resulting in no significant differences (*p* > 0.05) between the two pH levels.

Finally, the biomass formed during fermentation was analyzed (Fig. 2D). In this case, there is a clear difference between the biomass at pH 7 and pH 9, with the biomass being five times greater at pH 9.

The results achieved in this study for both pH are better than those reported in the literature. In this way, for example, Antonicelli et al. (2023) obtained lower concentrations of ethanol (0.4–0.7 g/L) and butanol (0.3–0.5 g/L), as well as acetic acid (0.9–1.0 g/L) and butyric acid (0.5–0.5 g/L) by *C. carboxidivorans* P7 (DSM 15243) from C1-gases mixtures such as H_2_:CO_2_ (4:1) and CO:H_2_:CO_2_:N_2_ (56:20:9:15). Lower levels of ethanol (0.9–1.0 g/L), butanol (0.2–0.4 g/L), acetic acid (0.6–0.7 g/L) and butyric acid (0.2–0.3 g/L) were also attained by *C. carboxidivorans* DSM 15243 from pure CO or the gases mixture CO:CO_2_:H_2_ (65:10:25) (Lanzillo et al. 2024).

Based on the analysis, although pH 9 results in higher biomass and alcohols production, this does not correlate with increased yields of organic acids. In addition, at pH 7, there is a more efficient consumption of CO (97.4 vs 61.8%), a higher overall production of acetic acid (1.2 vs 0.6 g/L) and a stable butyric acid production across fermentation. On the other hand, pH 7 is the optimal pH of C. beijerinckii, then could be also beneficial to work at this pH value in the co-culture by *C. carboxidivorans* and *C. beijerinckii*. Therefore, maintaining a natural initial pH (pH 7) could provide a suitable substrate utilization and subsequent formation of fermentation products, making it more effective for overall fermentation efficiency. In addition, the use of pH 7 do not involve the necessity to adjust this parameter with the consequent consumption of reagents, which could be beneficial for the process profitability.

#### *Influence of Fe*^*0*^

**Fig. 3:**
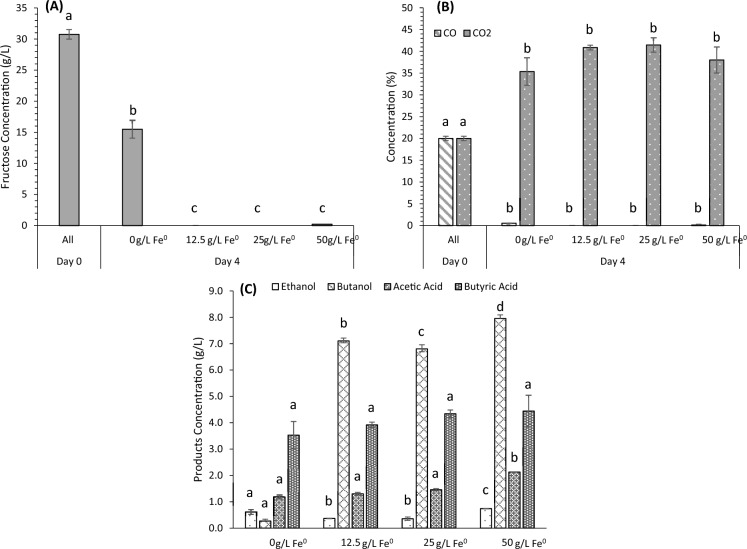
Fermentation co-culture results for different iron concentrations (0–12.5–25–50 g/L), by *C. carboxidivorans* (t = 0) and *C. beijerinckii* (t = 24h) at ratio 1:1 and pH_0_ = 7. **A** Fructose Concentration (g/L), **B** Gas Concentrations (CO and CO_2_) (%), and **C** Product Concentrations (Ethanol, Butanol, Acetic Acid, and Butyric Acid) (g/L). The results are at the 4th day related to the maximum product concentration obtained. Each letter denotes statistically significant differences for the corresponding compound

As previously discussed, the absence of H_2_ in the gas mixture used prevents the consumption of CO_2_. However, Bayar et al. (2022) suggest a more sustainable alternative to H_2_: using Fe^0^ as an electron donor for CO_2_ conversion. Fe^0^ presence, a potential electron donor for CO_2_ bioconversion, promotes the H_2_ generation under anaerobic conditions (Hwang et al. 2019; Bayar et al. 2022)), helping this to reduce CO_2_ to CO in the carbonyl branch of the WLP (Schuchmann and Müller 2014), which is metabolized by *C. carboxidivorans*. This approach could facilitate the consumption of CO_2_ by *C. Carboxidivorans* and potentially enhance the overall fermentation process. It is worth mentioning that Fe^0^ is not consumed nor dissolved during fermentation process, instead it is only used as a potential electron donor for CO_2_ bioconversion into CO. In this work, the effect of fermentation without the addition of Fe^0^ was compared against the addition of three different iron concentrations (12.5, 25, and 50 g/L). The results are presented in Fig. 3.

In relation to the substrate, Fig. 3A reveals the first difference in fructose consumption. Despite having previously observed that approximately half of the fructose was consumed at initial pH 7 (15 g/L), the presence of Fe^0^ into the fermentation medium, regardless of concentration, results in complete fructose consumption. On the other hand, regarding gas consumption (Fig. 3B), it is observed that both fermentations without Fe^0^ and those with Fe^0^ deplete all available CO in the headspace of the reactor. However, it is noteworthy that fermentations with Fe^0^ generate slightly more CO_2_ than those without iron, increasing from 35.5% to a range between 38% and 41.5%, with no significant differences among the four cases (*p* > 0.05). This could indicate that the addition of iron has positive effects as, despite doubling fructose consumption, which would normally lead to increased CO_2_ production, the observed increase in the headspace is at most 6 percentage points. Furthermore, the slight increase in CO_2_ production with iron addition may suggest a potential positive influence on the metabolic activity of microorganisms involved in the fermentation process. In fact, Bayar et al. (2022) found that, as fermentation progressed, CO_2_ production increased similarly in both iron-containing and iron-free fermentations. Additionally, they noted a significant increase in the products obtained in the iron-supplemented fermentations. On the other hand, it is worth highlighting that a considerable consumption of CO_2_ (about 60%) was achieved during the first days of fermentation by *C. carboxidivorans* using a mixture of CO:CO_2_:N_2_ (20:20:60) as a substrate and in the presence of Fe^0^ (50 g/L) (Figure S3).

Figure 3C illustrates the products obtained from these trials, with the primary distinction being the butanol concentration observed in fermentations with Fe^0^, increasing from 0.6 g/L to approximately 7–8 g/L. This concentration range is where *C. beijerinckii* reaches substrate inhibition. Moreover, it is worth mentioning that lower butanol concentrations (6.3 g/L) were achieved in cultures using only *C. beijerinckii*, similar to those reported in the literature using this same microorganism (Plaza et al. 2017).

On the other hand, examining other products obtained, for instance, ethanol remains relatively constant, below 1 g/L, with minor variations among the experiments (*p* > 0.05). However, the behavior of acids is particularly noteworthy. Both acetic acid and butyric acid show minimal differences between concentrations in experiments conducted with and without iron. Acetic acid ranges from 1.2 g/L (without Fe^0^) to 2.1 g/L (with 50 g/L of Fe^0^); while butyric acid varies from 3.5 g/L (without Fe^0^) to 4.4 g/L (with 50 g/L of Fe^0^), indicating an increase of approximately 1 g/L in both cases. This suggests that the addition of Fe^0^ enhances acid production in accordance with Bayar et al. (2022), possibly due to both microorganisms producing these acids during the acidogenic phase, and each utilizing a specific substrate, thereby fully consuming fructose, CO, and possibly CO_2_ in the presence of iron. Acid production naturally acidifies the medium, as the pH is uncontrolled. Additionally, it is known that *C. beijerinckii* can utilize acetic and butyric acids to produce butanol (Zhang et al. 2016b), strongly indicating the consumption of these acids in our study, leading to butanol levels ranging from 6.8 to 8 g/L.

When comparing these results with the literature, it can be observed that similar levels of butanol and butyric acid are achieved in fermentations using *C. beijerinckii* with waste substrates through the conventional process of pretreatment/enzymatic hydrolysis/fermentation. For instance, 8 g/L of butanol, 3.5 g/L of butyric acid, 0.5 g/L of ethanol, and 3.5 g/L of acetic acid were achieved by *C. beijerinckii* from sugarcane bagasse hydrolysates containing 40 g/L of total sugars (Vieira et al. 2021). Similarly, up to 6 g/L of butanol and 3.5 g/L of butyric acid were attained by *C. beijerinckii* using brewer’s spent grain hydrolysates with 28 g/L of glucose and 33 g/L of total sugars (Plaza et al. 2017). 6.7–8.0 g/L butanol were also obtained from enzymatic hydrolysates of carrot discard (López-Linares et al. 2023), microwave pretreated brewer’s spent grain (147 °C, 2 min and 1.26% H_2_SO_4_) (López-Linares et al. 2020) and microwave pretreated spent coffee grounds (160.47 ºC and 1.5% H_2_SO_4_) (López-Linares et al. 2021) by *C. beijerinckii* DSM 6422, using 100 mL serum bottles.

On the other hand, although the highest butanol concentration of 8 g/L was obtained with 50 g/L of Fe^0^ in the medium, showing significant differences (*p* < 0.05) compared to the other two Fe^0^ concentrations studied, the use of a lower Fe^0^ concentration (for instance, 12.5 g/L) could help to enhance the process feasibility, being able to achieve up to 7.1 g/L butanol. Therefore, the chosen Fe^0^ concentration to supplement the fermentation medium is 12.5 g/L.

These comparisons underscore the fact that the results obtained in this study are in line with established benchmarks, further validating the effectiveness of the mixotrophic fermentation process using *C. carboxidivorans* and *C. beijerinckii*. The ability to achieve comparable product yields using alternative substrates highlights the potential for sustainable and economically viable fermentation processes.

### *C. carboxidivorans* and *C. beijerinckii* co-cultures in bioreactor

Once the optimal working conditions for the co-culture of *C. carboxidivorans/C. beijerinckii* had been established, the process was scaled up to a stirred tank bioreactor. Two operational modes for the gas stream were considered: A) batch and B) continuous. This approach aimed to investigate how these formats affect the fermentation process.

**Fig. 4:**
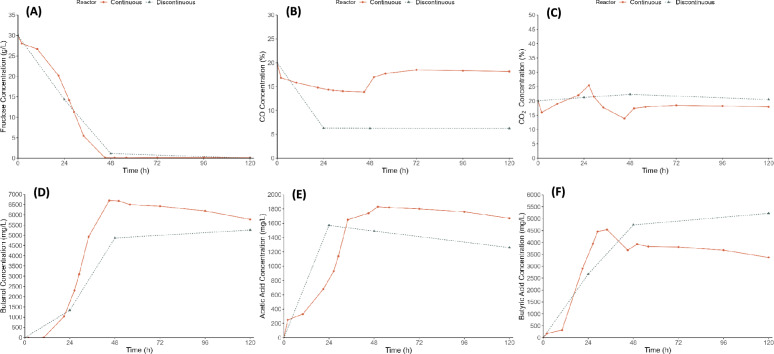
Co-culture mixotrophic fermentation in bioreactor with discontinuous or continuous (50 mL/min) feeding gas (fructose: 30 g/L and CO/CO_2_: 20%/20%), by *C. carboxidivorans* (t = 0) and *C. beijerinckii* (t = 24h) at ratio 1:1, 12.5 g/L of Fe^0^ and initial pH 7*.* **A** Fructose (g/L), **B** CO (%), **C** CO_2_ (%), **D** Butanol (g/L), **E** Acetic Acid (g/L), **F** Butyric Acid (g/L)

Figure 4 presents the results obtained for both configurations. As shown in Fig. 4A, fructose is completely consumed for both gas feeding strategies within approximately the same time frame (48 h). This indicates that the availability of the primary carbon source is not significantly impacted by the gas addition mode. Figure 4B and C illustrate the gas addition profiles for CO and CO_2_, respectively. For CO, Fig. 4B shows that, in the DSTR configuration, there is an 80% reduction in CO consumption initially, followed by a plateau, indicating that *C. carboxidivorans* ceases to consume CO after the initial phase. Conversely, with the continuous gas feeding, there is a continuous decrease in the CO concentration at the reactor outlet, suggesting ongoing dissolution and consumption of CO by *C. carboxidivorans*. However, after 48 h, when the fructose is depleted in the CSTR, the CO concentration at the outlet stabilizes at the initial input percentage (~ 20%). This fact could be due to *C. carboxidivorans* stops metabolizing CO after 48 h of process, reaching its maximum production of butanol, and acetic and butyric acids. Similarly, for CO_2_, Fig. 4C indicates that, in the discontinuous gas feeding configuration, the CO_2_ percentage remains relatively constant, most likely because all the CO_2_ produced during fermentation dissolves in the fermentation broth, then becomes to CO thanks to H_2_ generated by Fe^0^ presence, and finally consumed by *C. carboxidivorans*. This behavior is different to those observed in bottles fermentation (Fig. 3), were CO_2_ increased, being both processes carried out under the same fermentation conditions (30 g/L fructose, CO/CO_2_/N_2_: 20%/20%/60%, ratio 1:1 *C. carboxidivorans* (t = 0):*C. beijerinckii* (t = 24h), 12.5 g/L Fe^0^ and initial pH 7). This fact could be due to the different agitation system used in the bioreactor (where a turbine is used for agitation, unlike in bottles in orbital shaker), which could stimulate the action of Fe^0^ in the liquid phase, then promoting the H_2_ generation and consequently helping to reduce CO_2_ to CO, then no being CO_2_ accumulated in discontinuous bioreactor (batch mode) (Fig. 4) unlike that in bottles fermentation (Fig. 3). In this context, Hazeena et al. (2019) also reported considerable differences in bioreactor fermentations compared with shake flasks. In contrast, the continuous gas feeding shows a slight increase in CO_2_ at the reactor outlet; implying that the microorganism cannot consume all the CO_2_ available in the continuous gas stream entering the reactor. This is evidenced by a 25% CO_2_ concentration at 24 h, indicating that some CO_2_ is carried by the gas stream without dissolving or being consumed. However, between 24 and 48 h, there is a decrease (25% to 15%) in CO_2_, probably due to the presence of Fe^0^ in the liquid phase that promotes the H_2_ generation and then, helps to reduce CO_2_ to CO.

After 48 h, the CO_2_ stream stabilizes around 15%, indicating that fermentation has concluded, and the microorganisms have ceased activity. The comparison of the two operational modes suggests that the continuous gas feeding may promote a better gas dissolution and utilization, potentially enhancing the overall fermentation efficiency. However, the discontinuous mode might be more effective in ensuring complete consumption of the gas substrates during the initial fermentation phase. Moreover, the differences observed in gas consumption patterns between the two modes could be attributed to the gas–liquid mass transfer dynamics and the microbial uptake rates. The continuous gas flow most likely provides a more stable and uniform distribution of gases, enhancing their availability for microbial consumption. On the other hand, the discontinuous gas feeding may create periods of gas limitation and excess, affecting the overall efficiency of the gas uptake. Additionally, it is important to consider the metabolic state of the microorganisms. In the discontinuous operation, the initial high consumption of CO followed by a plateau might indicate a shift in the metabolic pathways of *C. carboxidivorans* from active CO consumption to maintenance or stress responses due to the depletion of readily available carbon sources. On the opposite, the continuous availability of CO could maintain the microorganisms in an active metabolic state, leading to more consistent gas consumption rates. In addition, it is worthing noting that, as can be reported from the literature (Fernández-Naveira et al. 2016; Vees et al. 2022), C1-gases fermentation processes usually are carried out using the continuous gas feeding strategy.

The fermentation results indicate the presence of butanol, acetic acid, and butyric acid. Considering that the substrate consumption in the continuous gas feeding strategy concludes at 48 h, the comparison focuses on this time point, achieving a butanol production of 7.0 g/L; whereas the discontinuous gas feeding strategy yields 4.6 g/L (Fig. 4D), indicating that the continuous gas supply positively impacts the fermentation process. This continuous supply likely enhances the availability of substrates, improving overall fermentation efficiency. In terms of acid production, a peak acetic acid concentration of approximately 1.8 g/L (at 48 h), which then remains stable, was obtained when the gas was continuously fed; whereas, around 1.6 g/L (at 24 h) was produced when the discontinuous gas feeding was used (Fig. 4E). However, the butyric acid production scenario is different. When the discontinuous gas feeding strategy was studied, butyric acid reached a maximum of 5.0 g/L (at 48 h) (Fig. 4F). On the other hand, the continuous gas feeding reached up to 4.5 g/L at 24 h, with a slight decrease to 4.0 g/L related to the increase in butanol concentration after 24 h of fermentation. This decrease and subsequent stabilization suggest that *C. beijerinckii* may be consuming the acids produced by *C. carboxidivorans* and converting them into butanol, further enhancing the butanol yield in the continuous gas feeding strategy. Comparing these results, the continuous gas feeding strategy not only enhances butanol production, but also affects the dynamics of acids produced during fermentation. The stabilization of acetic acid concentration and the decrease in butyric acid in the continuous gas feeding strategy could be linked to the efficiency of the co-culture in utilizing these by-products for butanol synthesis. Thanapornsin et al. (2022) achieved higher butanol concentrations (10.1–10.6 g/L) but much lower butyric acid levels (< 1 g/L) from sugarcane molasses by *C. beijerinckii* TISTR 1461, using stirred-tank and gas-lift bioreactors.

**Fig. 5:**
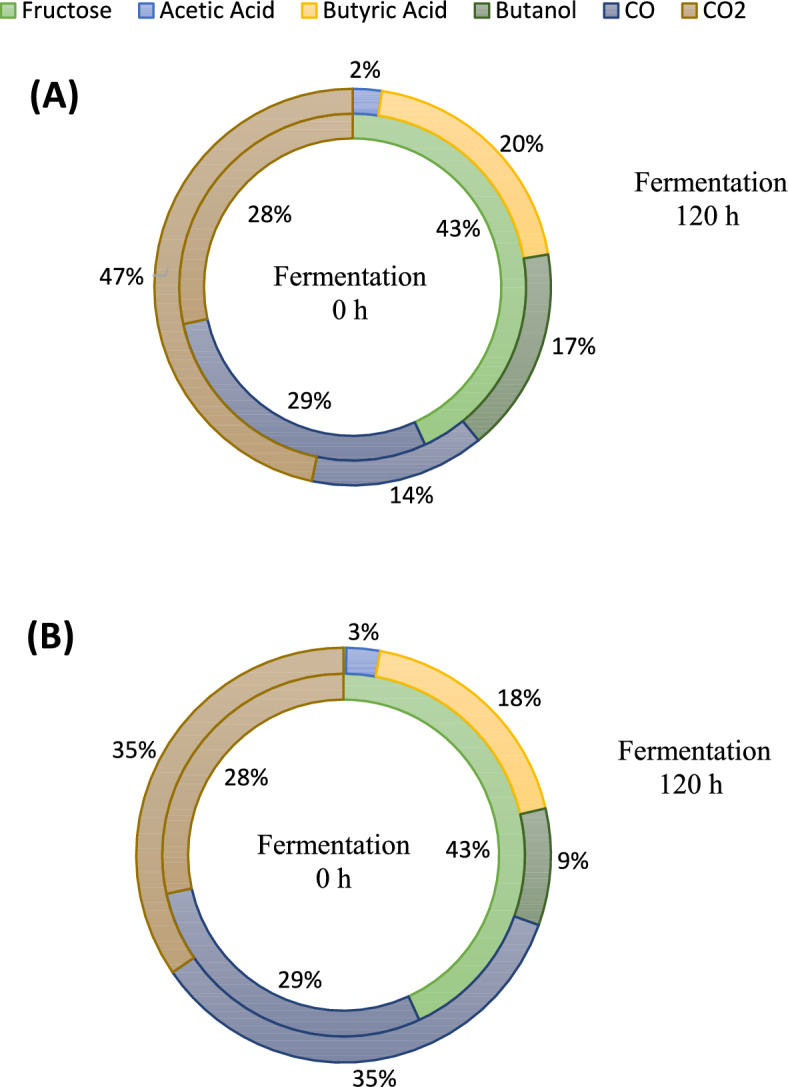
Carbon balance of co-culture mixotrophic fermentation in bioreactor with discontinuous (**A**) or continuous (50 mL/min) (**B**) feeding gas (fructose: 30 g/L and CO/CO_2_: 20%/20%), by *C. carboxidivorans* (t = 0) and *C. beijerinckii* (t = 24h) at ratio 1:1, 12.5 g/L of Fe^0^ and initial pH 7. The inner circle represents time 0 h, and the outer circle represents 120 h

Figure 5 compares the evolution of carbon from the initial compounds (CO/CO_2_/fructose) over 120 h of fermentation for the two reactor configurations studied. In both cases, the initial carbon distribution consists of 43% from fructose, and 29%/28% from CO/CO_2_. However, the final carbon distribution differs between the two reactors, as expected. In the discontinuous gas feeding strategy (Fig. 5A), there is complete fructose consumption, and the CO content decreases from 29 to 14%. The carbon from the reactants is transformed into final products: 2% acetic acid, 20% butyric acid, and 17% butanol, alongside an increase in CO_2_ from 28 to 47%. Conversely, in the continuous gas feeding strategy, the final carbon distribution differs, mainly due to the constant gas addition to the reactor. With complete fructose consumption, the relative carbon content of CO and CO_2_ increases to 35%. Regarding the fermentation products, the relative proportions are 3% acetic acid and 18% butyric acid (both similar to the discontinuous gas feeding strategy), and 9% butanol (half of that in the discontinuous gas feeding strategy).

## Conclusions

This study investigates the fermentation of *C. carboxidivorans* in a mixotrophic co-culture with *C. beijerinckii*, analyzing the influence of the microorganism ratio, pH and Fe^0^ addition. The mixotrophic co-culture showed synergy between the species, improving the production of ethanol, acetic acid, and butyric acid as compared to individual fermentations. A microorganism ratio of 1:1 at pH 7 and 12.5 g/L of Fe^0^ improved butanol production, reaching up to 7 g/L. Scaling up to a stirred tank bioreactor compared discontinuous and continuous gas feeding strategies, with better gas utilization and fermentation efficiency observed in continuous mode. However, further investigation on long-term stability, operational costs, and potential challenges for large-scale applications of mixotrophic co-cultures fermentation by *C. carboxidivorans* and *C. beijerinckii* is needed.

## Supplementary Information

Additional file1.

## Data Availability

The datasets used in the current study are available from the corresponding author on reasonable request.
