# Supplementary material for: Bioconversion of C1-gases by mixotrophic co-cultures fermentation with C. carboxidivorans and C. beijerinkii
Source: Bioresour Bioprocess. 2025 May 26;12(1):45. doi: 10.1186/s40643-025-00881-w (PMC12104125; doi:10.1186/s40643-025-00881-w)
Supplement: Supplementary file 1 — Additional file1. [file 40643_2025_881_MOESM1_ESM.docx]

Figure S1. *C. carboxidivorans* *heterotrophic fermentation:* Concentration of fructose (g/L) (A), biomass OD (B), and production of ethanol (g/L) (C), acetic acid (g/L) (D), and butyric acid (g/L) (E) at pH from 5 to 9. Error bars represent standard deviation from average experimental results.

**
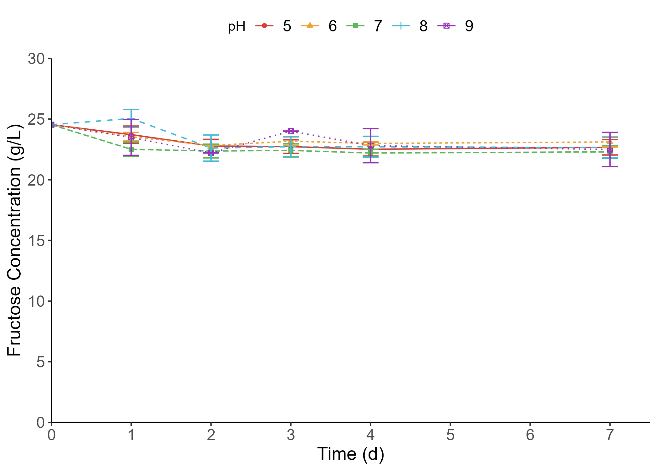

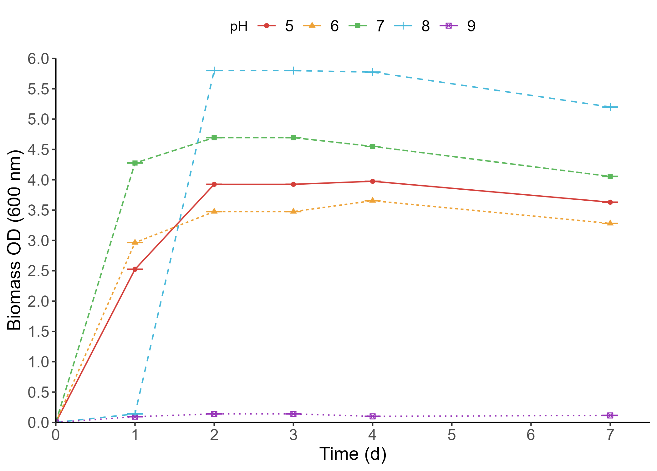
**

**(C)**

**(B)**

**(A)**

**
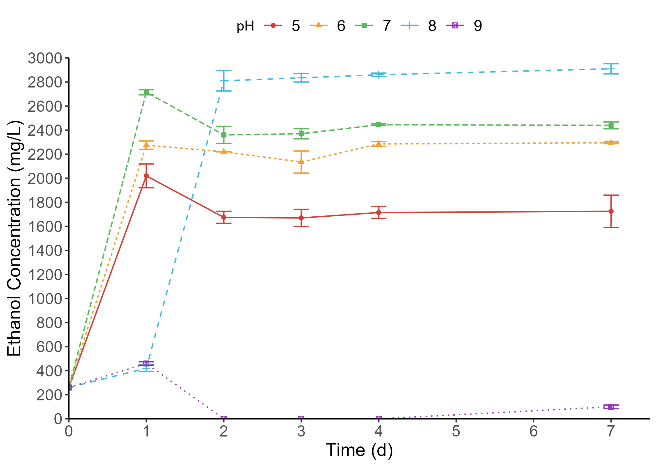

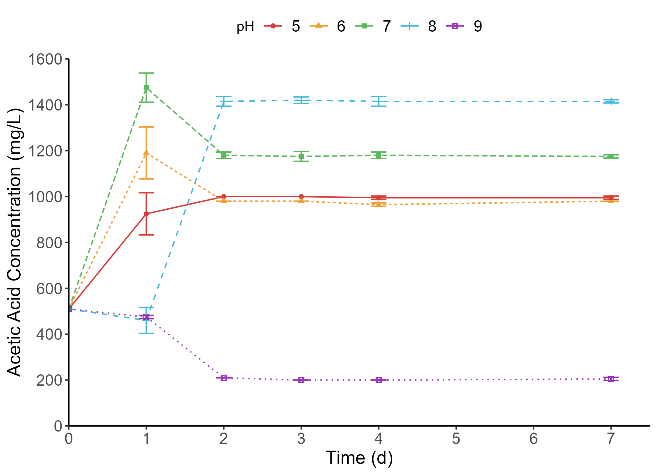

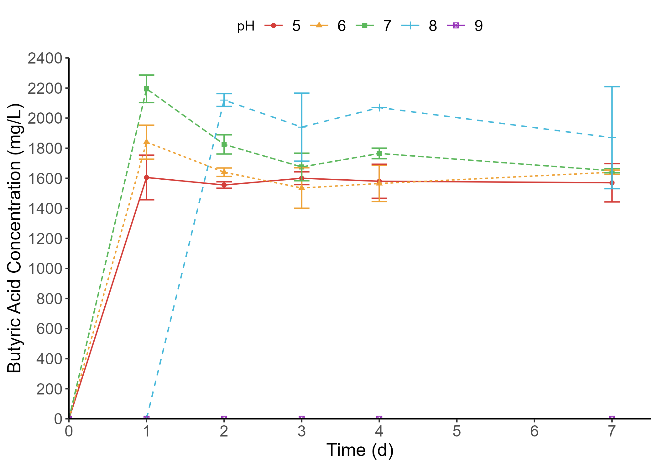
**

**(E)**

**(D)**

Figure S2. *C. carboxidivorans* *autotrophic fermentation:* Concentration of CO (%) (A) and CO_2_ (%) (B), biomass OD (C), and production of ethanol (g/L) (D), acetic acid (g/L) (E), and butyric acid (g/L) (F) at pH from 5 to 9. Error bars represent standard deviation from average experimental results.

**
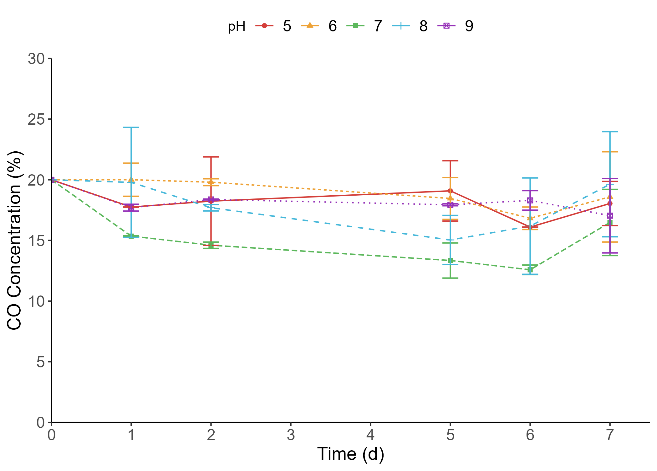

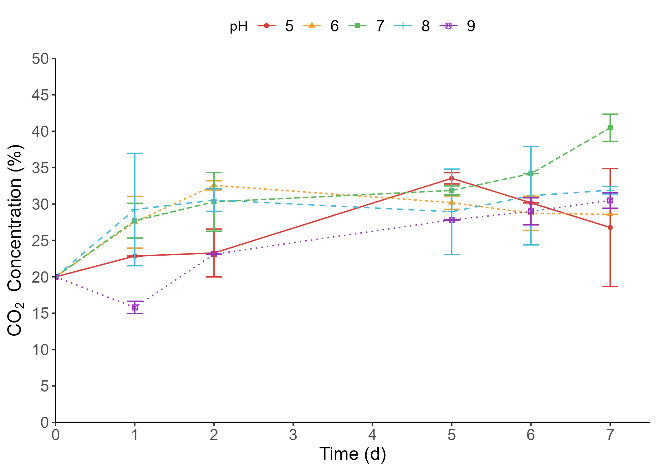

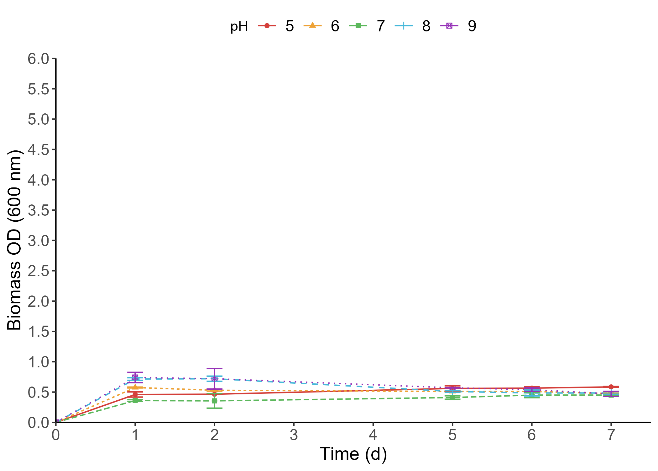

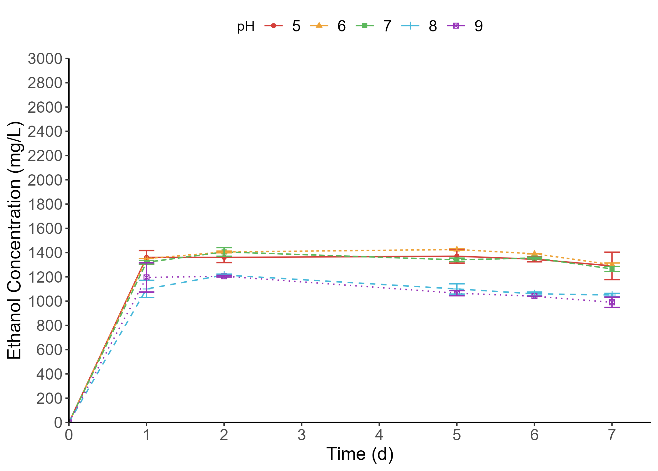
**

**(D)**

**(C)**

**(B)**

**(A)**

**(F)**

**(E)**


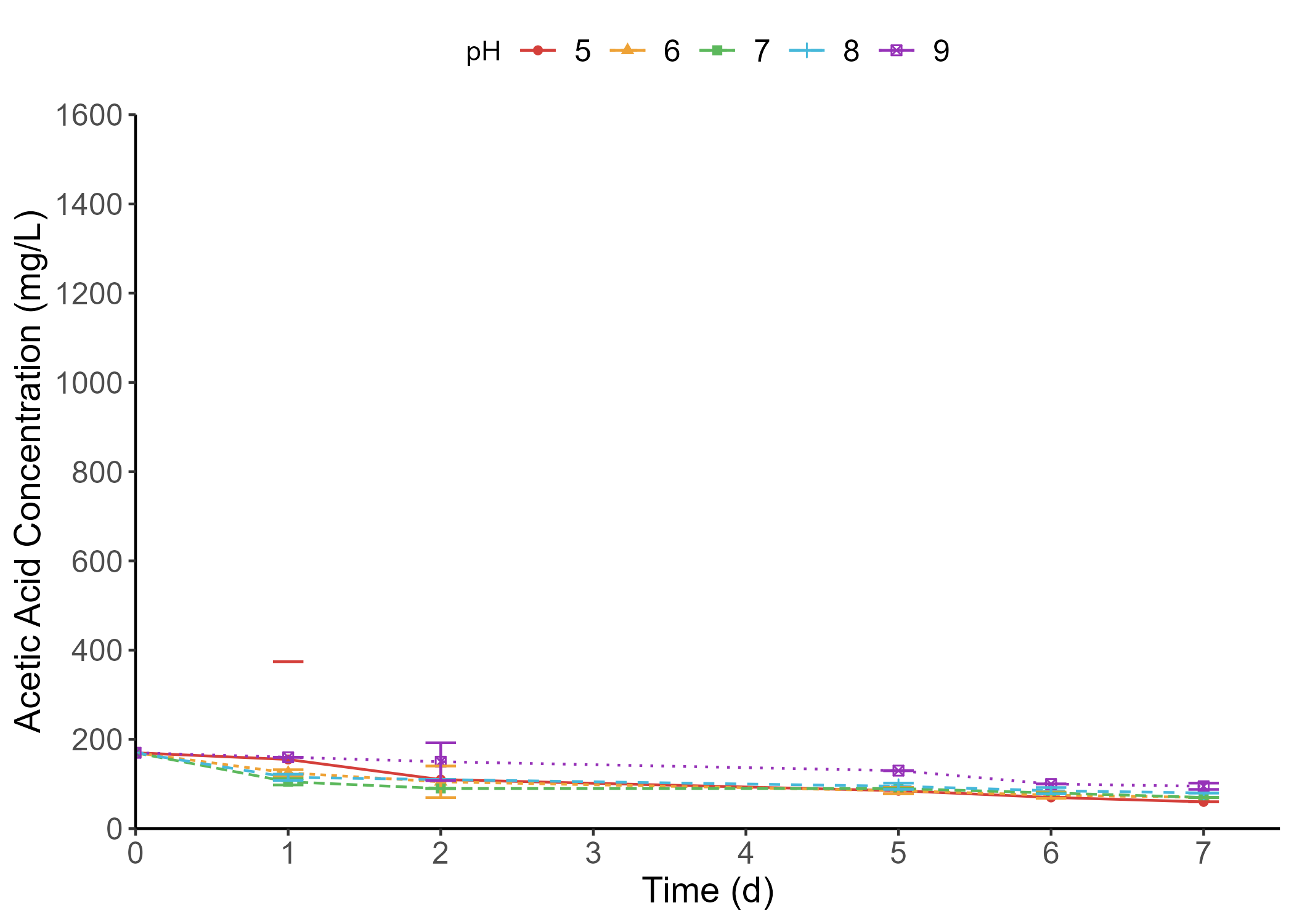

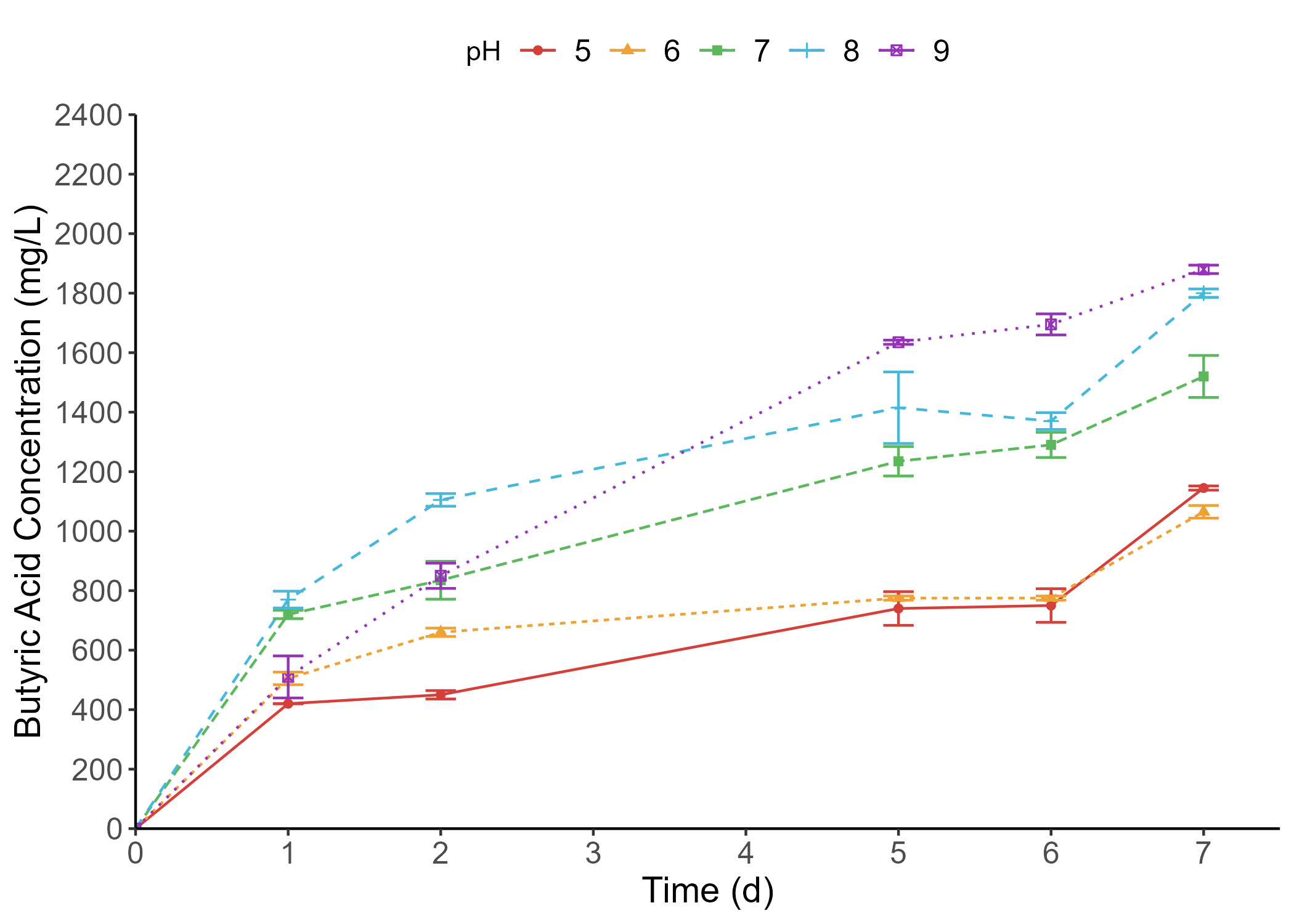


Figure S3. Profile of CO (A) and CO_2_ (B) concentrations (%) during autotrophic fermentation by *C. carboxidivorans*, at pH 6 and in presence of 50 g/L of Fe^0^ (CO/CO_2_: 20%/20%).

**(A)**

**(B)**
